# REPURPOSED FOR AGING

**DOI:** 10.1093/geroni/igae098.0449

**Published:** 2024-12-31

**Authors:** Sandra Aleksic

**Affiliations:** Chair

## Abstract

Chronic diseases represent the leading driver of health care cost, with detrimental impact on function, independence, and quality of life in the aging society. Geroscience-guided approaches seek to target aging biology to extend healthspan, defined as the period of life spent without major disease and disability. Repurposing Food and Drug Administration (FDA) - approved drugs as gerotherapeutics (geroscience-guided pharmacologic interventions) offers the advantage of known pharmacologic profile and existing clinical experience and can represent a cost-effective strategy to test interventions for extension of healthspan. Among FDA-approved drugs, candidates with geroscience potential can be identified based on the evidence of extension of healthspan and lifespan in model organisms coupled with clinical evidence of benefits extending beyond the diseases targeted by the drug, through mechanisms that involve modulation of hallmarks of aging. Given the inseparable connections between metabolism and aging, it is not surprising that several FDA-approved drugs developed for treatment of metabolic dysfunction are emerging as potential gerotherapeutics. Metformin (discussed by Dr. Sara Espinoza), a widely used oral antidiabetic drug, extends lifespan in preclinical models, while observational clinical evidence supports its role in prevention of several chronic diseases. Two newer antidiabetic drug classes, glucagon-like peptide 1 (GLP1) receptor agonists (discussed by Dr. John Newman) and sodium- glucose cotransporter 2 (SGLT2) inhibitors (discussed by Dr. Carolina Solis-Herrera), have demonstrated healthspan and lifespan benefits above and beyond treatment of diabetes. Emerging evidence for modulation of multiple hallmarks of aging supports gerotherapeutic properties of these drugs. Clinical trials targeting several aging-relevant outcomes are underway.

